# Development and Plasticity of Cognitive Flexibility in Early and Middle Childhood

**DOI:** 10.3389/fpsyg.2017.01040

**Published:** 2017-06-20

**Authors:** Frances Buttelmann, Julia Karbach

**Affiliations:** ^1^Department of Psychology, Goethe University FrankfurtFrankfurt, Germany; ^2^Center for Research on Individual Development and Adaptive Education of Children at Risk (IDeA),Frankfurt, Germany; ^3^Department of Developmental Psychology, Friedrich Schiller University JenaJena, Germany; ^4^Department of Psychology, University of Koblenz-LandauLandau, Germany

**Keywords:** cognitive flexibility, intervention, childhood, executive functions, metacognition, DCCS, task switching

## Abstract

Cognitive flexibility, the ability to flexibly switch between tasks, is a core dimension of executive functions (EFs) allowing to control actions and to adapt flexibly to changing environments. It supports the management of multiple tasks, the development of novel, adaptive behavior and is associated with various life outcomes. Cognitive flexibility develops rapidly in preschool and continuously increases well into adolescence, mirroring the growth of neural networks involving the prefrontal cortex. Over the past decade, there has been increasing interest in interventions designed to improve cognitive flexibility in children in order to support the many developmental outcomes associated with cognitive flexibility. This article provides a brief review of the development and plasticity of cognitive flexibility across early and middle childhood (i.e., from preschool to elementary school age). Focusing on interventions designed to improve cognitive flexibility in typically developing children, we report evidence for significant training and transfer effects while acknowledging that current findings on transfer are heterogeneous. Finally, we introduce metacognitive training as a promising new approach to promote cognitive flexibility and to support transfer of training.

## Introduction

Cognitive flexibility, the ability to shift between different tasks or goals, is considered a key aspect of executive functions (EF) allowing individuals to regulate their thoughts and actions adaptively (e.g., Miyake et al., 2000; Jurado and Rosselli, 2007). In the literature, it is also referred to by shifting, attention switching, or task switching, and includes both the ability to disengage from irrelevant information in a previous task and to focus on relevant information in a forthcoming task (Monsell, 2003). Thus, cognitive flexibility enables to think divergently, change perspective and adapt to a continuously changing environment.

When it comes to the structure of EF, earlier models have either assumed that it is a unitary construct (e.g., Duncan et al., 1997) or a set of dissociable control components (e.g., Stuss and Alexander, 2000). More recent approaches have shown the unity and diversity of EF in integrative frameworks (e.g., Miyake et al., 2000; Garon et al., 2008). The Miyake model, for instance, assumes that the core EF skills entail working memory (WM), inhibitory control, and cognitive flexibility. Importantly, this structure is subject to developmental changes, with a shift from a single latent EF factor to separate component processes from early childhood to school age and adolescence (e.g., Huizinga et al., 2006; Wiebe et al., 2008, 2011).

Importantly, EF in general and cognitive flexibility in particular contributes to a number of important life outcomes, such as academic achievement (review: Titz and Karbach, 2014). Colé et al. (2014), for instance, showed that cognitive flexibility predicted reading skills in second graders and a recent meta-analysis showed that cognitive flexibility was a significant predictor for both math and reading skills in children between the ages of 4 and 13 years (Yeniad et al., 2013). Given the strong relationship between flexibility and achievement, it is not surprising that many studies have aimed at training flexibility in order to improve children’s performance in the classroom (review: Titz and Karbach, 2014; meta-analysis: Schwaighofer et al., 2015). We will focus on such training effects in the last section of this review. In the upcoming section, we will first describe the development of cognitive flexibility.

## Development of Cognitive Flexibility

Infants within their first year of life already exhibit fundamental forms of EF (Carpenter et al., 1998), but the core components (WM, inhibition and flexibility; Miyake et al., 2000) rapidly develop during the preschool years (Hughes, 1998). Research focusing on the development across the lifespan demonstrates that EF continues developing throughout childhood (e.g., Davidson et al., 2006) well into adolescence (e.g., Huizinga and van der Molen, 2007) and early adulthood (e.g., Anderson et al., 2001). In this review, however, our focus will be on the preschool and elementary-school age. We will illustrate developmental changes in flexibility by referring to two widely used paradigms assessing children’s cognitive flexibility, the *Dimensional Change Card Sort* task (DCCS; Zelazo, 2006) and the *task-switching paradigm* (Monsell, 2003).

Most studies investigating preschoolers applied the DCCS to test cognitive flexibility. In this task, children are shown cards with pictures displaying two dimensions (e.g., color and shape) and are told to sort these cards by one dimension (e.g., by color) (pre-switch phase). At some point, participants are told to sort the cards by the other dimension (i.e., by shape) (post-switch phase). While children from the age of 4 years are able to switch the rules, 3-years-old typically perseverate and keep applying the first rule when they should apply the second one (e.g., Zelazo, 2006; Doebel and Zelazo, 2015). Performance continues to improve with age, as children are able to apply higher-order rules and handle more complex tasks (e.g., Chevalier and Blaye, 2009; Diamond, 2013), such as the task-switching paradigm. In this task, children are instructed to perform two tasks (A and B), e.g., two simple categorization tasks. In single-task blocks, participants perform both tasks separately (e.g., AAA, BBB), but in mixed-task blocks, they have to switch between both tasks (e.g., AABBAABB). This paradigm allows assessing two different components of cognitive flexibility – the ability to switch from one rule/task to another as well as the maintenance and selection of task sets in WM. Karbach and Kray (2007) tested 5- to 6-years-old and 9-years-old on a cued task-switching paradigm. In task A, children had to categorize stimuli as either fruits or animals and in task B they had to indicate if the picture was presented in color or gray. Results showed an age-related improvement in the ability to maintain and select tasks, but not in the ability to switch between tasks. These different developmental trajectories of the processes subserving cognitive flexibility were confirmed by other studies applying switching tasks and investigating a wider range of ages (e.g., Cepeda et al., 2001; Crone et al., 2004; Reimers and Maylor, 2005; Huizinga and van der Molen, 2007; Kray et al., 2008). For instance, Huizinga and van der Molen (2007) examined the developmental change in switching and maintenance and found that children reached adult levels of switching abilities by the age of 11 years, while task maintenance abilities only matured at the age of 15 years. In sum, these findings point to an earlier maturation of task-switching than task-maintenance and selection abilities.

Developmental trajectories of EF are have been linked to maturational changes of the prefrontal cortex (PFC) and associated cortical and subcortical structures, including parietal regions and basal ganglia (e.g., Casey et al., 2005; Bunge and Wright, 2007). Some regions within the PFC, such as the orbitofrontal cortex, reach structural maturity at an earlier age, whereas others, such as the dorsolateral PFC, show a more protracted maturational time course (Gogtay et al., 2004). There is evidence – including studies using the DCCS and the task-switching paradigm – suggesting that those differences in structural maturation are paralleled by changes in functional maturation and hence may account for distinct developmental trajectories among EFs (Bunge and Zelazo, 2006).

For instance, a study by Moriguchi and Hiraki (2009) assessed 3- and 5-year-old children as well as adults with the DCCS task using NIRS (near-infrared spectroscopy). Results for the 3-years-old indicated that only some 3-years-old who passed the task showed significant activation in the right inferior PFC. In contrast, 5-years-old and adults showed this activation bilaterally (see also Moriguchi and Hiraki, 2014). This finding was consistent with another longitudinal study (Moriguchi and Hiraki, 2011) testing children at the age of 3 and 4 years. In contrast to age 3, children at age 4 passed the task and showed an increasing activation in the left inferior PFC (cf. Morton et al., 2009). Together with the finding that functional connectivity between the lateral PFC and inferior parietal cortex increases as children age (Ezekiel et al., 2013), these findings add to the evidence indicating that the PFC is a key player in the development of cognitive flexibility.

Studies using a task-switching paradigm confirm these age differences in brain activation. Rubia et al. (2006), for instance, found age-related increases in the recruitment of several brain regions that have been implicated in cognitive flexibility, including right inferior PFC, left parietal cortex, anterior cingulate cortex (ACC), and striatum. Moreover, there is neuroscientific evidence supporting the different developmental trajectories of task switching and task maintenance/selection: Crone et al. (2006) tested children, adolescents and adults and found an adult-like pattern of activation for task switching in the pre-supplementary motor area by adolescence. In contrast, the activation for task maintenance and selection in the ventrolateral PFC differed among children, adolescents, and adults (see Wendelken et al., 2012, for similar patterns of activation in children and adults, but different timing, pointing more to a change in the temporal dynamics rather than qualitative changes during development).

Taken together, the behavioral and neuroimaging results demonstrate that cognitive flexibility rapidly increases during early and middle childhood, suggesting that this may be a period of high plasticity and malleability sensitive to developmental as well as environmentally driven changes. It is not surprising then that much research focused on interventions designed to support the development of EF. These interventions range from school and curriculum-based programs to physical and cognitive training regimes (for reviews see Diamond, 2012; Karbach and Unger, 2014).

## Plasticity of Cognitive Flexibility – Training and Transfer Effects

When it comes to training of EF, most of the existing developmental studies have certainly targeted WM (for reviews see Könen et al., 2016; Rueda et al., 2016). However, there are a handful of studies training cognitive flexibility in early and middle childhood. While some have trained multiple components of EF at the same time (e.g., Röthlisberger et al., 2012; Traverso et al., 2015), others have focused specifically on cognitive flexibility. We will illustrate this line of research by reviewing interventions applying the DCCS and the task-switching paradigm. We will report training effects and also evidence for transfer of training-related gains to untrained tasks and abilities, which recently has been discussed very controversially in the community (e.g., Shipstead et al., 2012).

Kloo and Perner (2003) trained 3- and 4-year-old children on the DCCS. Before and after training, the children performed the DCCS and a false-belief task (as well as a number of control tasks) including a novel version of the DCCS with different test and target cards at post-test. Children in the DCCS training group showed larger improvements on the DCCS and the false-belief task than children in the control group. They also outperformed the control group on the novel DCCS task. Thus, training did not only benefit cognitive flexibility but also transferred to false-belief understanding. Also training DCCS performance, van Bers et al. (2014) studied the effects of feedback on cognitive flexibility in 3-years-old. Providing feedback on the post-switch sorting improved DCCS performance compared to a standard condition without feedback. Importantly, these gains transferred to a novel version of the DCCS administered immediately after training as well as 1 week later.

In school-aged children, a number of studies have applied the task-switching paradigm to train cognitive flexibility. Adopting a lifespan approach, Cepeda et al. (2001) tested a sample ranging from 7–82 years of age on single-task and mixed-task blocks (*N* = 152). After three sessions of training, participants – and particularly children – significantly improved task maintenance and selection (Kray et al., 2008).

Following up on these training gains, other studies investigated whether task-switching training also transfers to untrained tasks and domains (e.g., Karbach and Kray, 2009; Zinke et al., 2012). Karbach and Kray (2009) had children (8–10 years of age) as well as younger and older adults (*N* = 168) perform four sessions of task-switching training. Results showed that training improved performance in an untrained switching task compared to a control group performing single-task training. Further, training also improved inhibition, verbal and visuo-spatial WM and fluid intelligence. Based on the transfer to WM and inhibition, another study tested the effects of task-switching training in children with ADHD because they usually show significant deficits in these domains. And indeed, four sessions of switching training resulted in significant improvements in an untrained switching task, inhibition and WM in 7- to 12-year-old boys with ADHD (*N* = 20; Kray et al., 2012).

These findings indicate that training cognitive flexibility may be a key factor for improving other dimensions of EF. Still, it has to be noted that transfer was less pronounced in other studies: Zinke et al. (2012) assessed the effects of task-switching training in10- to 14-years-old (*N* = 80). After three sessions of training, participants showed significant training gains and also transfer to an untrained switching task, but no transfer to inhibition. These effects mirror data from 8- to 11-years-old performing task-switching training embedded in a game environment (Dörrenbächer et al., 2014).

Thus, training regimes based on the DCCS and task-switching yielded significant improvements in cognitive flexibility across childhood and adolescence. Moreover, there is evidence showing that they can result in transfer to other EF dimensions, even though results on transfer of switching training are heterogeneous, just as they are for other types of cognitive training (for reviews, see Karbach and Kray, 2016; Könen et al., 2016). However, the existing studies almost exclusively analyzed data on the group level and ignored individual differences in training-induced gains. Given that even individuals participating in exactly the same training regime usually highly differ in their training outcomes (for reviews see Könen and Karbach, 2015; Katz et al., 2016), it is crucial to study individual differences in baseline performance as well as the individual performance development during training to understand these differential outcomes. Previous studies, for instance, showed that EF training often resulted in compensation effects, indicating that participants with lower baseline performances benefitted more (e.g., Cepeda et al., 2001; Bherer et al., 2008; Karbach and Kray, 2009; Zinke et al., 2012) and that individual differences in age and fluid intelligence (Bürki et al., 2014), motivational aspects (Katz et al., 2016), and the amount of training gain (e.g., Jaeggi et al., 2011) contributed to the success of training interventions. However, the underlying mechanisms are still largely unknown, especially in early childhood.

Another aspect that gains more and more attention in the field of training research is the question which aspects of intervention designs moderate training-induced gains. While current meta-analyses have tested effects related to the intensity, frequency and adaptivity of training, just to name a few (e.g., Karbach and Verhaeghen, 2014; Au et al., 2015; Schwaighofer et al., 2015), other features – such as the instructional design of training – have received less attention. However, since EF entails higher-level cognitive processes, it has been proposed that metacognitive processes, i.e., reflecting on one’s own thinking and actions, may be important for the development and plasticity of EF (e.g., Zelazo et al., 2003; Chevalier and Blaye, 2016). This aspect has been investigated in a few recent studies. Espinet et al. (2013) showed across three experiments that training with corrective feedback and instruction to reflect on the task led to substantial improvements in DCCS performance in 2- to 4-years-old. Compared to controls, trained children benefitted more on an untrained version of the DCCS. Moreover, they showed a significant reduction of the N2 amplitude (an indicator of conflict detection) during DCCS performance and at the same time an increase in reaction time. The authors concluded that slowing down may have provided the time needed to reflect on the hierarchical nature of the DCCS task and to resolve the conflict inherent in the task (Espinet et al., 2012).

Similarly, Moriguchi et al. (2015) trained 3- to 5-year-old preschoolers on the DCCS in two experiments. Children performed a pre-test, training and a post-test. In the experimental group, they interacted with a puppet and were asked to explain the task with all the rules to the puppet, to think about task demands or possible strategies to solve the task in order to foster metacognitive reflection. Results showed that the experimental group improved from pre-test to post-test and performed significantly better than the control group at post-test. Moreover, using NIRS Moriguchi et al. (2015) showed a higher activation in the left PFC after training, again confirming the importance of the PFC for EF.

There is also evidence from task switching: Chevalier and Blaye (2016) investigated whether children’s EF monitoring drives EF development from 6 to 10 years of age. They recorded gaze position while participants performed a self-paced task-switching paradigm. In this task, the children had as much time as they needed to proactively prepare for the next task. Both the analysis of gaze trajectories and performance showed that older children were better prepared than younger ones when they responded, even though younger participants could have taken more time to prepare their response. Thus, with increasing age children are better able to monitor EF engagement, pointing to the important contribution of metacognitive processes to EF development.

Even though these findings highlight the importance of metacognition for efficient EF functioning, metacognitive instructions have rarely been applied in cognitive-training research. Unlike many previous training approaches, metacognitive EF training would not aim to enhance the quantity of EF that children can engage, but to change qualitatively how they engage EF as a function of task difficulty (for an example of metacognitive training in reasoning research see Houdé et al., 2000, 2001). Thus, metacognitive training should facilitate the flexible adaptation to new tasks by training the children to reflect on how to approach them, for instance integrating information about current task demands and past experiences in order to weigh the respective costs (e.g., mental effort) and benefits (e.g., rewards) of available control strategies (cf. Chevalier and Blaye, 2016). Metacognitive training should further encourage performance evaluation, including error detection and feedback processing, all of which are still gradually developing in young children (e.g., Chevalier et al., 2009; Andersen et al., 2014; DuPuis et al., 2014). Given that this metacognitive approach is relatively task-unspecific, it may even support transfer of flexibility training to untrained tasks and abilities. Future studies may want to consider this promising approach when designing new interventions to improve cognitive flexibility (or EF in general).

## Conclusion

Cognitive flexibility develops rapidly during the preschool years and continues to improve across adolescence and young adulthood. Given that EF, and cognitive flexibility in particular, are related to many important life outcomes including academic achievement (e.g., mathematics or reading skills; Yeniad et al., 2013; Titz and Karbach, 2014) and even health status during adulthood (Moffitt et al., 2011), numerous interventions have been designed to improve childhood EF.

Recent training studies provided accumulating evidence for the trainability of cognitive flexibility in early and middle childhood. We illustrated these training effects and also findings on transfer based on studies applying the DCCS and the task-switching paradigm. Training on both tasks has been shown to transfer to other dimensions of EF and to core dimensions of theory of mind, such as false-belief understanding. Importantly, these effects were not only present on the behavioral level but also mirrored by eye-tracking and neuroscientific measures. Given that the mechanisms underlying these training and transfer effects are not fully understood, future studies should try disentangling them, possibly by considering individual differences in training outcomes and by testing the role of metacognitive processes in the plasticity of cognitive flexibility in childhood.

## Author Contributions

All authors listed have made a substantial, direct and intellectual contribution to the work, and approved it for publication.

## Conflict of Interest Statement

The authors declare that the research was conducted in the absence of any commercial or financial relationships that could be construed as a potential conflict of interest.
